# House dust mite sensitization drives cross-reactive immune responses to homologous helminth proteins

**DOI:** 10.1371/journal.ppat.1009337

**Published:** 2021-03-02

**Authors:** Pedro Henrique Gazzinelli-Guimaraes, Sasisekhar Bennuru, Rafael de Queiroz Prado, Alessandra Ricciardi, Joshua Sciurba, Jonah Kupritz, Matthew Moser, Olena Kamenyeva, Thomas B. Nutman

**Affiliations:** 1 Laboratory of Parasitic Diseases, NIAID, National Institutes of Health; Bethesda, Maryland, United States of America; 2 Biological Imaging Section of Research Technologies Branch, National Institutes of Health; Bethesda, Maryland, United States of America; University of Medicine & Dentistry New Jersey, UNITED STATES

## Abstract

The establishment of type 2 responses driven by allergic sensitization prior to exposure to helminth parasites has demonstrated how tissue-specific responses can protect against migrating larval stages, but, as a consequence, allow for immune-mediated, parasite/allergy-associated morbidity. In this way, whether helminth cross-reacting allergen-specific antibodies are produced and play a role during the helminth infection, or exacerbate the allergic outcome awaits elucidation. Thus, the main objective of the study was to investigate whether house dust mite (HDM) sensitization triggers allergen-specific antibodies that interact with *Ascaris* antigens and mediate antibody-dependent deleterious effects on these parasites as well as, to assess the capacity of cross-reactive helminth proteins to trigger allergic inflammation in house dust mite presensitized mice. Here, we show that the sensitization with HDM-extract drives marked IgE and IgG1 antibody responses that cross-react with *Ascaris* larval antigens. Proteomic analysis of *Ascaris* larval antigens recognized by these HDM-specific antibodies identified *Ascaris* tropomyosin and enolase as the 2 major HDM homologues based on high sequence and structural similarity. Moreover, the helminth tropomyosin could drive Type-2 associated pulmonary inflammation similar to HDM following HDM tropomyosin sensitization. The HDM-triggered IgE cross-reactive antibodies were found to be functional as they mediated immediate hypersensitivity responses in skin testing. Finally, we demonstrated that HDM sensitization in either B cells or FcγRIII alpha-chain deficient mice indicated that the allergen driven cell-mediated larval killing is not antibody-dependent. Taken together, our data suggest that aeroallergen sensitization drives helminth reactive antibodies through molecular and structural similarity between HDM and *Ascaris* antigens suggesting that cross-reactive immune responses help drive allergic inflammation.

## Author summary

Epidemiological studies related to the interaction between allergies and helminth infection led to the observations that helped shape the so-called hygiene hypothesis, which generally states that chronic exposure to helminths diminishes the risk of the development of allergic disease. However, there are conflicting studies that have called this particular hypothesis into question, such as, the studies that suggest that infection with the helminth *Ascaris lumbricoides* is a risk factor for wheezing and atopy or can aggravate the clinical symptoms of asthma. A hypothetical explanation for such phenomenon is the fact that there is a high degree of molecular and structural similarities among helminth antigens with many common allergens, including the house dust mite (HDM). This high degree of homology of certain epitopes shared between helminths and allergens generate cross-react antibodies which may play a role in the pathogenesis or regulation of both conditions. Thus, this study aimed to understand the structural basis for cross-reactive antibodies induced by HDM sensitization. Here, we demonstrate that HDM sensitization drives helminth cross-reactive antibodies through molecular and structural homology between tropomyosins and enolases. This study highlights the pro-allergenic properties of HDM and helminth proteins that share homologous epitopes.

## Introduction

Over the years, many studies have examined the interface between allergic diseases and helminth infections in situations when they coexist. In the majority of allergy/helminth infection studies reported to date, the acquisition of helminth infections was felt to precede the onset of atopy [1–3]. In these studies, the data largely demonstrated that chronic helminth infection modulates the allergic response through the induction of helminth-induced IL-10, expansion of regulatory T cells and blocking IgG4 antibodies [4–6]. There are, however, studies in humans and in experimental models that have called into question this particular corollary to the “hygiene hypothesis,” most notably the studies demonstrating that *A*. *lumbricoides* infection is associated with asthma and wheezing [7–10]. Other studies have shown that *Clonorchis sinensis* infection is positively associated with atopy [11]. In this regard, it has been suggested that relatively acute helminth infection promotes type-2-associated immune polarization and IgE sensitization that, because of the structural similarities in B-cell epitopes between helminths and allergens, drive allergen-specific cross-reactive antibodies [12–14].

More recently, and based on immunological observations in humans [15] and in experimental models [16], it has been shown that allergic sensitization prior to acquisition of a helminth infection drives an eosinophil-rich type 2 immune response that has consequences for both pathology in the lung and for parasite development [16]. Indeed, this allergen-driven eosinophil-dominated type 2 response in the lungs led to a marked reduction in the number of *Ascaris* lung-stage larvae and to a profound developmental arrest in those larvae that survived. Moreover, it has been demonstrated that *Ascaris*-driven IgG leads to protection against the larval ascariasis (early larval migration) in experimental infections in mice, indicating an important role for antibodies in controlling parasite burden [17]. However, further studies need to be addressed to investigate whether HDM-specific responses contribute to the eosinophil-dominated type-2 inflammatory response responsible for the lung-specific larval helminth killing.

The main objective of the study was to understand the structural basis for cross-reactive antibodies induced by HDM sensitization and how this understanding sheds new light on the allergy/helminth interactions. Our data not only provide evidence for the importance of these cross-reactive helminth antigens, but also identify the major parasite antigens/allergens responsible for the cross-reaction/cross-sensitization.

## Results

### Pulmonary pre-sensitization with HDM-allergens drives a marked systemic antibody response that cross-react *in vitro* with helminth *Ascaris* antigens

**Fig 1:**
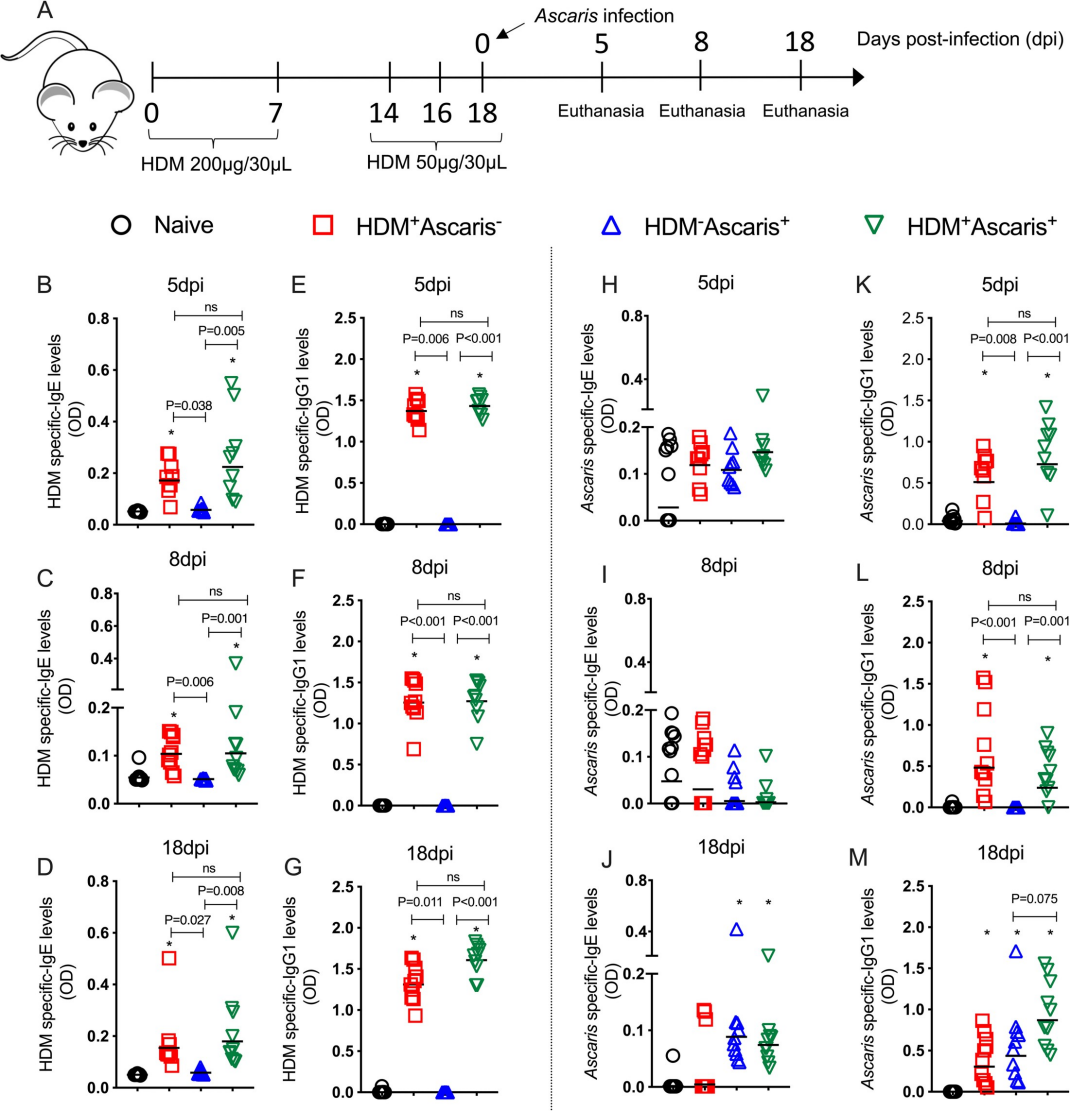
HDM-allergic sensitization drives a marked IgE and IgG1 antibody response that cross-react with helminth *Ascaris* antigens. Experimental design scheme for HDM-allergic sensitization followed by *Ascaris* infection in Balb/c mice (A). For the x axis, we used the number of days post infection with *Ascaris* (5, 8 and 18 dpi that also represent the number of days after the fifth and last sensitization with house dust mite extract. HDM-specific IgE (B-D) and IgG1 (E-G) and *Ascaris*-specific IgE (H-J) and IgG1 (K-M) levels were measured at day 5 (n = 9 mice/group), day 8 (n = 10 mice/group) and day 18 (n = 10 mice/group) in the sera of non-allergic and non-infected naive mice [HDM^-^*Ascaris*^-^ (**Ο**)]; allergic but not infected mice [HDM^+^*Ascaris*^-^ (◻)]; non-allergic but infected mice [HDM^-^*Ascaris*^+^ (Δ)]; and, allergic and infected mice [HDM^+^*Ascaris*^+^ (∇)]. Each symbol represents a single mouse and the horizontal bars are the geometric means. P values are indicated in each graph. Kruskal-Wallis test followed by Dunn’s Multiple Comparison Test was used for all comparisons and * indicates significantly different (p<0.05) from naïve (HDM^-^Ascaris^-^) group.

To understand the role of HDM-induced antibodies in the allergen driven type-2 protective immune response to the parasite larvae [16], we examined the HDM-specific and *Ascaris*-specific antibody production over time (Fig 1). Our data show that the HDM-sensitization induced strong systemic HDM-specific IgE levels (Fig 1B–1D) and HDM-specific IgG1 levels (Fig 1E–1G) in the HDM-sensitized groups (HDM^+^*Ascaris*^-^ and HDM^+^*Ascaris*^+^) at all-time points evaluated. In addition, and as expected, the HDM- unsensitized mice (HDM^-^*Ascaris*^*+*^ and the naïve HDM^-^*Ascaris*^-^) had no HDM-specific IgE or IgG1 antibodies.

The assessment of *Ascaris*-specific IgE and IgG1 levels in the context of the larval ascariasis without sensitization with HDM allergens revealed no differences in antibody levels at 5- and 8-dpi between non-allergic but infected (HDM^-^*Ascaris*^+^) and naïve mice. In contrast, at 18 dpi, parasite-driven IgE and IgG1 responses in the HDM^-^*Ascaris*^+^ mice could be seen. Most interestingly, animals sensitized with HDM but not infected with *Ascaris*, (HDM^+^*Ascaris*^*-*^) demonstrated a marked increase in the *Ascaris*-specific IgG1 levels, at 5-, 8- and 18-days following allergic sensitization (Fig 1K–1M), suggesting that there is significant cross-reactivity between HDM-specific antibodies and antibodies to structurally related *Ascaris* proteins.

### HDM-driven antibodies recognize antigens on the surface of early *Ascaris* migrating larval-stages

**Fig 2:**
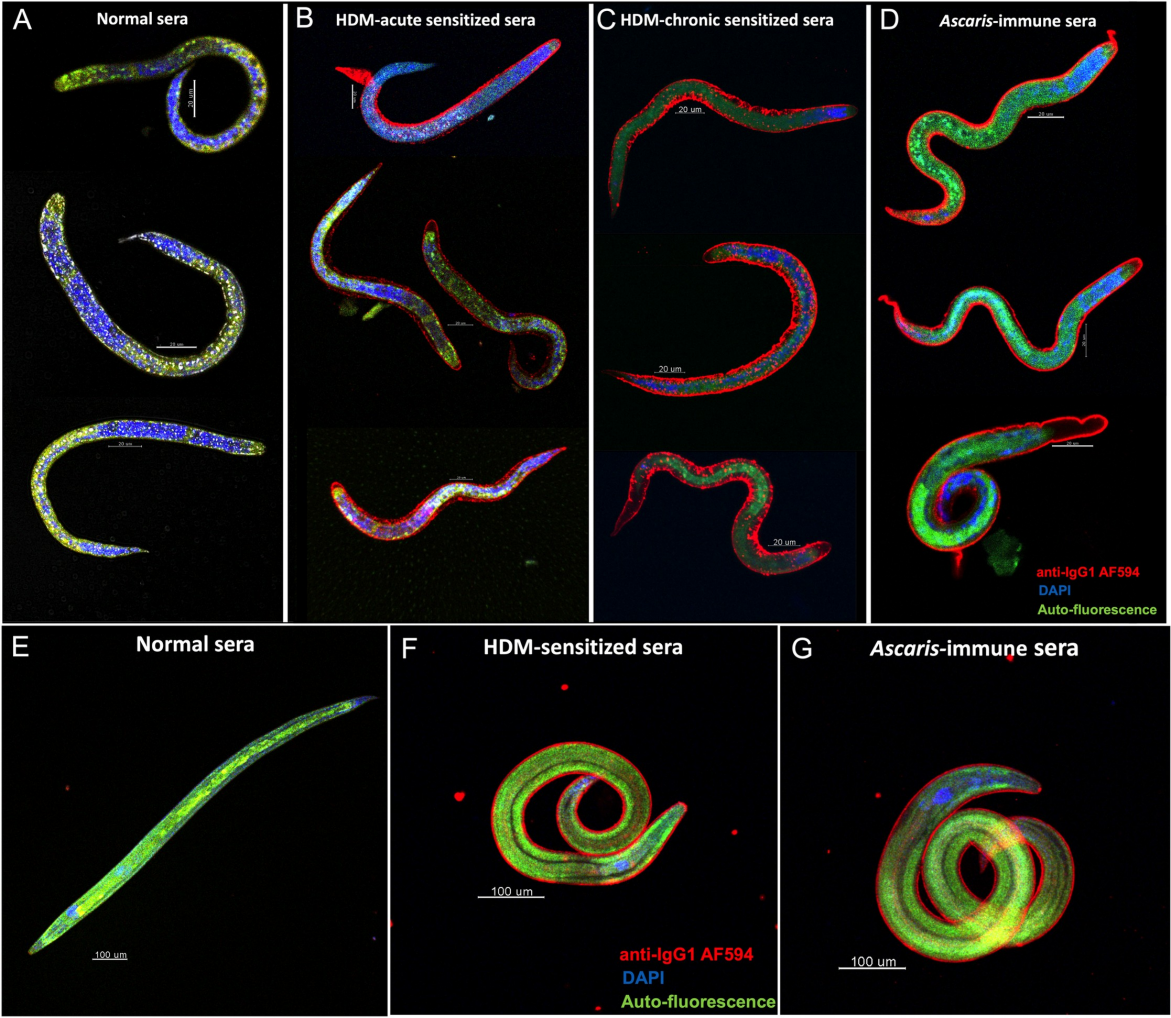
HDM-driven antibodies recognize antigens in the surface of early *Ascaris* migrating larval-stages. Representative confocal microscopy images highlighting the HDM-specific IgG1 (red) with the infective L3 larvae (A-D) and lung-stage larvae (E-G) using sera from naïve mice (A and E), HDM-sensitized mice (B, C and F), and *Ascaris*-immune sera (D and G). Nuclear staining with DAPI (blue) and autofluorescence (green) is also seen.

We next tested if HDM-driven IgG1 antibodies would bind to the surface of migrating *Ascaris* larval stages using confocal microscopy (Fig 2). As seen, serum from the HDM-sensitized mice stained the surface of both the infective L3 larvae (Fig 2B) and the lung-stage larvae (Fig 2F) to a similar degree to that seen from the serum pool from *Ascaris*-infected mice (Fig 2D and 2G). In contrast, sera from naïve mice failed to recognize antigens on the larval stages.

Interestingly, chronic sensitization with HDM (additional HDM intranasal sensitizations) demonstrated higher levels of cross-reactive IgG1 antibodies (S1C Fig), as well as, stronger recognition of the antigens on the surface of the larval stages (Fig 2C). Although HDM-specific IgE levels were very abundant in HDM sera, there was little helminth cross reactive IgE when assessed by ELISA (S1D Fig) or confocal microscopy (S2 Fig).

### Antigenic targets of *Ascaris* lung-stage larvae that cross-react with HDM-specific antibodies

**Fig 3:**
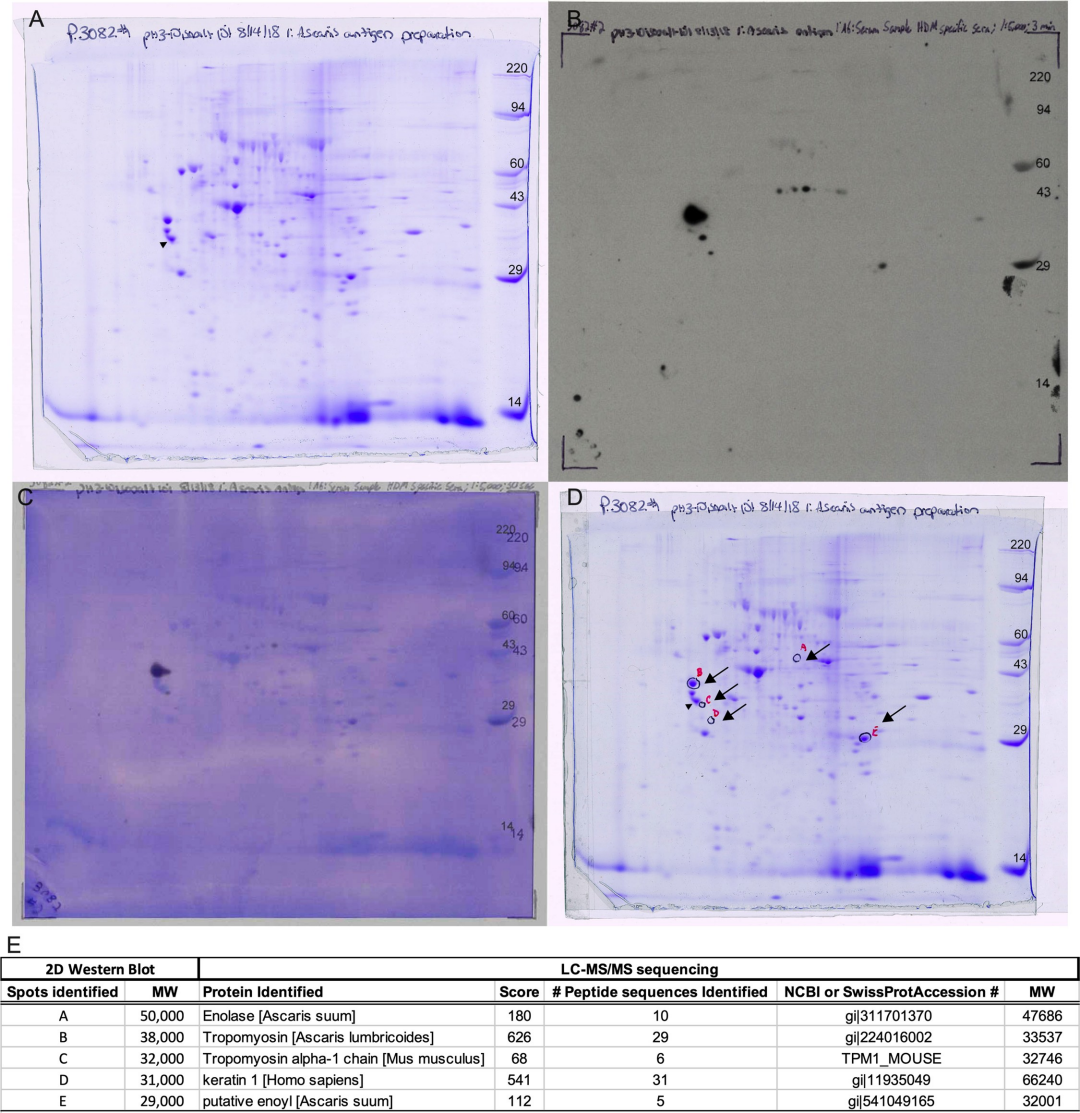
Proteomics profile of *Ascaris* lung-stage larval antigens that cross-react with HDM-driven antibodies. Coomassie blue-stained 2-D SDS-PAGE gel of *Ascaris* lung-stage larval antigens (A). Immunoblot of lung-stage larval crude antigen using a serum pool of HDM-allergic BALB/c mice (B) and the reactive bands (arrows) in the gel for protein excision based on the overlay (C) of Panels A and B (D). Results of the LC-MS sequencing of the A-E excised spots showing the protein identification and their respective molecular weight, based on the peptide sequence similarities using NCBI All Organisms, NCBI Mammalia & SwissProt All Organisms databases (E).

To identify and characterize the potential *Ascaris*-encoded antigen targets of the HDM-specific antibodies, we used 2-dimensional gel electrophoresis (2D SDS-PAGE) and immunoblotting (Fig 3). There was HDM-driven antibody reactivity to 9 lung-stage larval proteins (Fig 3B), of which 5 appeared to be recognized by the cross-reactive antibodies (Fig 3D and 3E- left column). These 5 antigens were excised, and LC-MS/MS analysis was performed; 3 (A, B and E) were confirmed unequivocally to be *Ascaris* proteins. The two remaining spots (C and D) were found to be mouse and human proteins, respectively. Thus, the 3 major *Ascaris* proteins found were *Ascaris* enolase (spot A), *Ascaris* tropomyosin, (spot B) and *Ascaris* enoyl-CoA hydratase putative protein (spot E).

**Fig 4:**
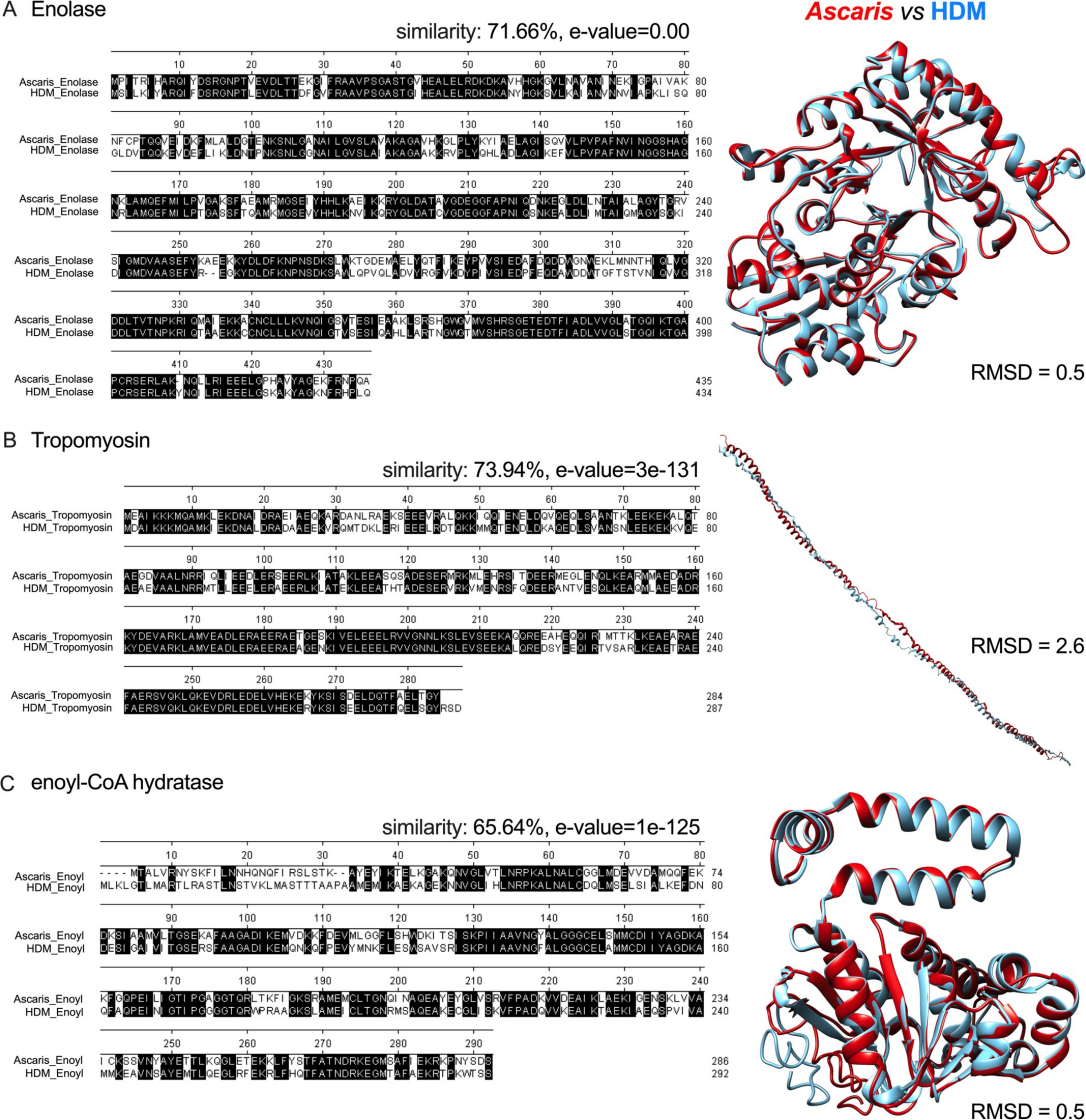
Protein sequences of the antigenic targets from lung-stage *Ascaris* larvae that cross-react with HDM-driven antibodies and their molecular and structural level of similarity with HDM homolog proteins. Alignment of Ascaris and HDM enolase (A), tropomyosin (B) and enoyl-CoA hydratase (C) sequences showing identical (shaded in black) amino acids and predicted 2D structure for *Ascaris* (red) and HDM (blue).

Sequence analyses and predicted 3D structural models indicated a high level of homology between *Ascaris* and HDM enolase (Fig 4A; 71.66% similarity, p = 0.00), *Ascaris* and HDM tropomyosin (also known as Der p 10) (Fig 4B; 73.94% similarity, p = 3e^-131^) and, *Ascaris* and HDM enoyl-CoA hydratase (Fig 4C; 65.64% similarity, p = 1e^-125^). Furthermore, analysis of the 3D structural similarity also revealed overlapping structures for *Ascaris* enolase, tropomyosin and enoyl-CoA-hydratase, with their HDM-equivalent proteins (RMSD = 0.5 angstroms; 2.6 angstroms, and 0.5 angstroms, respectively).

**Fig 5:**
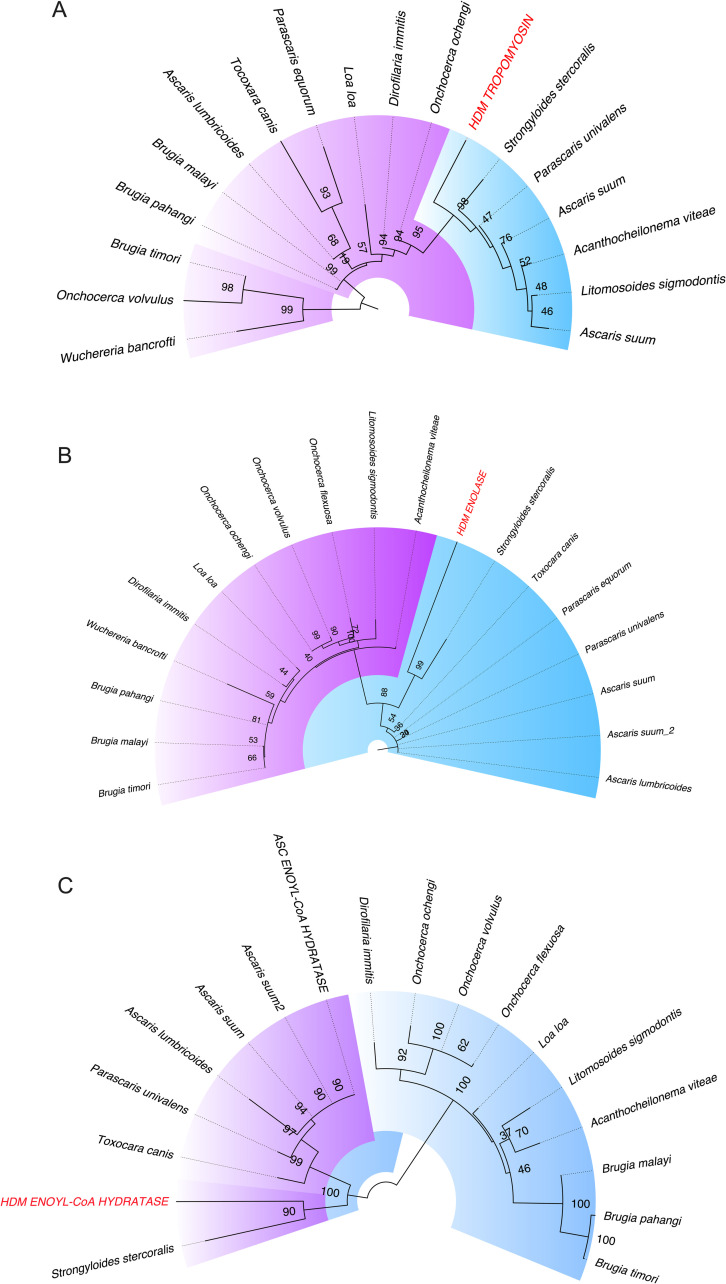
Phylogenetic trees highlighting the evolutionary relationships between house dust mite and helminth proteins. Alignments of tropomyosin (A), enolase (B) and enoyl-CoA-hydratase (C) with their helminth orthologues indicating their level of distance and phylogeny. Different clades are separated by colors. Two different proteomic datasets *from Ascaris suum* were used for the analysis.

To expand the observations of the nature of this homology, we have constructed phylogenetic trees to demonstrate that these proteins have orthologues among many different helminth parasites. The evolutionary analysis revealed a clear conserved link between house dust mite tropomyosin (Fig 5A) enolase (Fig 5B) and enoyl-CoA-hydratase (Fig 5C) with different helminth parasite nematodes of public health importance, including the filarial parasites *Wuchereria bancrofti* and *Brugia* sp. (lymphatic filariasis), *Loa loa* loiasis and *Onchocerca volvulus* (onchocerciasis- river blindness). Moreover, as can be seen, HDM tropomyosin, enolase and enoyl-CoA-hydratase segregate in the same clade with those from other soil-transmitted helminths (*Toxocara canis*, *Ascaris* sp., and *Strongyloides stercoralis*).

### HDM-specific antibodies recognize lung-stage *Ascaris* recombinant tropomyosin and enolase

**Fig 6:**
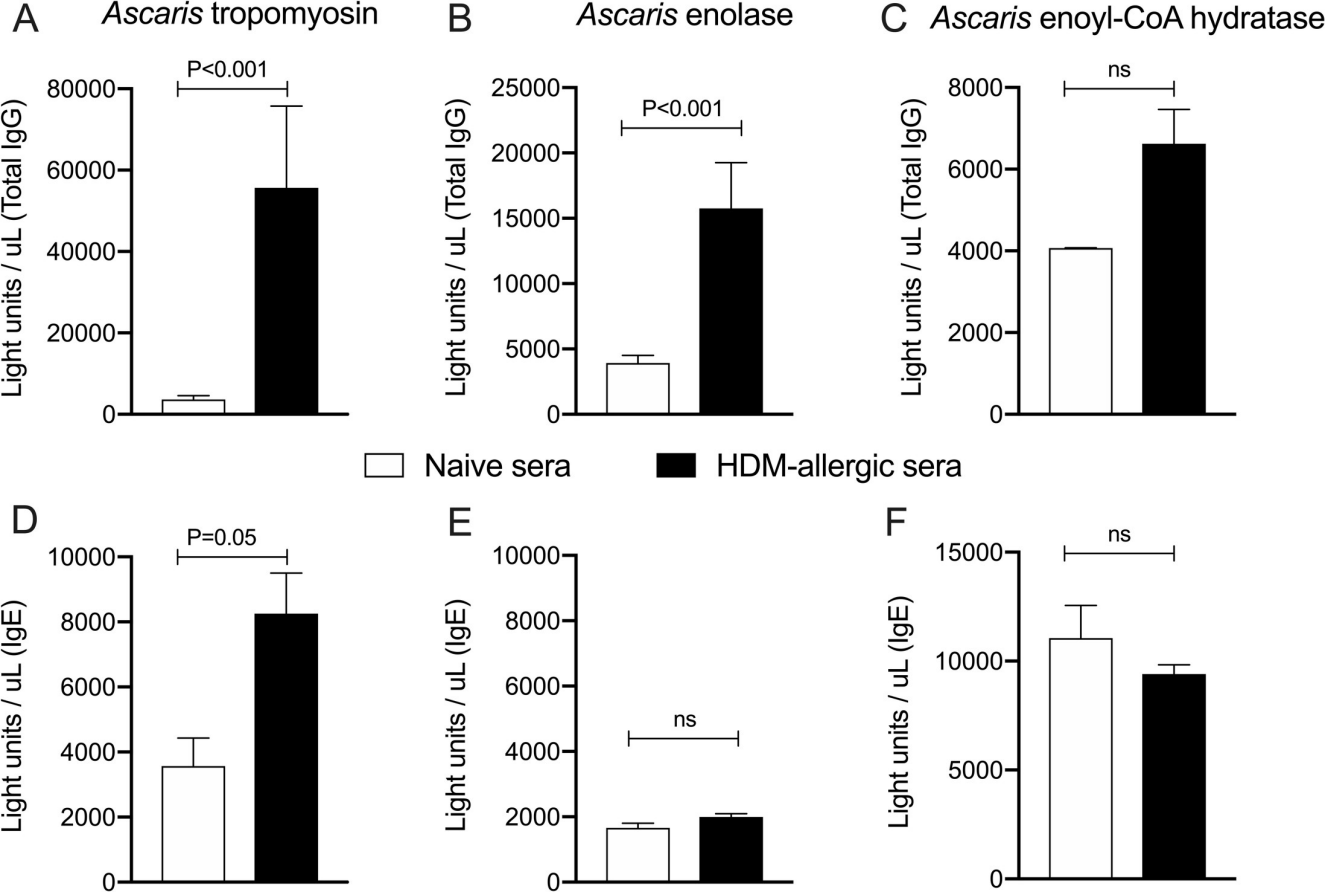
HDM-allergic IgG1 and IgE recognize lung-stage *Ascaris* recombinant proteins. LIPS assay showing the HDM-driven IgG (A, B, C) and IgE (D, E, F) reactivity to *Ascaris*-encoded tropomyosin (A-D), enolase (B-E) and enoyl-CoA hydratase (C-F). Data are expressed as light units/uL. Bars represent the Mean + SEM of five pools of sera with 5 mice each from HDM-allergic mice and naïve mice. P values are indicated.

The specificity of the cross-reactive HDM-antibodies (IgG and IgE) to *Ascaris* tropomyosin, enolase and enyol-coA hydratase proteins was assessed by LIPS. As can be seen in Fig 6, there was clear recognition of *Ascaris* enolase and *Ascaris* tropomyosin (55,671 ± 20,071 RLU and 15,762 ± 3,503 RUL, respectively) by the sera from HDM-sensitized mice that differed significantly from that seen in the naive mice [3,674 ± 929.9 light units/uL (P<0.001) and 3,928 ± 581.4 light units/uL (P<0.001), respectively] (Fig 6A and 6B). *Ascaris* enoyl-CoA hydratase was not recognized differentially by sera from HDM-allergic mice compared to sera from naïve mice [6,625 ± 835.7 light units/uL vs. 4,070 ± 70.5 light units/uL (P>0.05), respectively] (Fig 6C), suggesting that *Ascaris* tropomyosin and enolase are the two major targets for the cross-reactive antibodies.

Unlike the IgG responses, significant IgE responses could be observed only for tropomyosin when sera from HDM-sensitized mice was compared with sera from naive mice (8,257 ± 1,244 lights unit/uL and 3,570 ± 862.2 lights unit/uL respectively, P = 0.05) (Fig 6D). *Ascaris* enolase and enoyl-CoA hydratase recombinant proteins were not recognized by IgE from HDM-sensitized mice (Fig 6E and 6F).

### Homologous helminth proteins exacerbate pulmonary allergic inflammation in house dust mite presensitized mice

After characterizing these set of homologous proteins between HDM and *Ascaris* parasites, we assessed the functional consequences of these cross-reactive proteins/antibodies.

**Fig 7:**
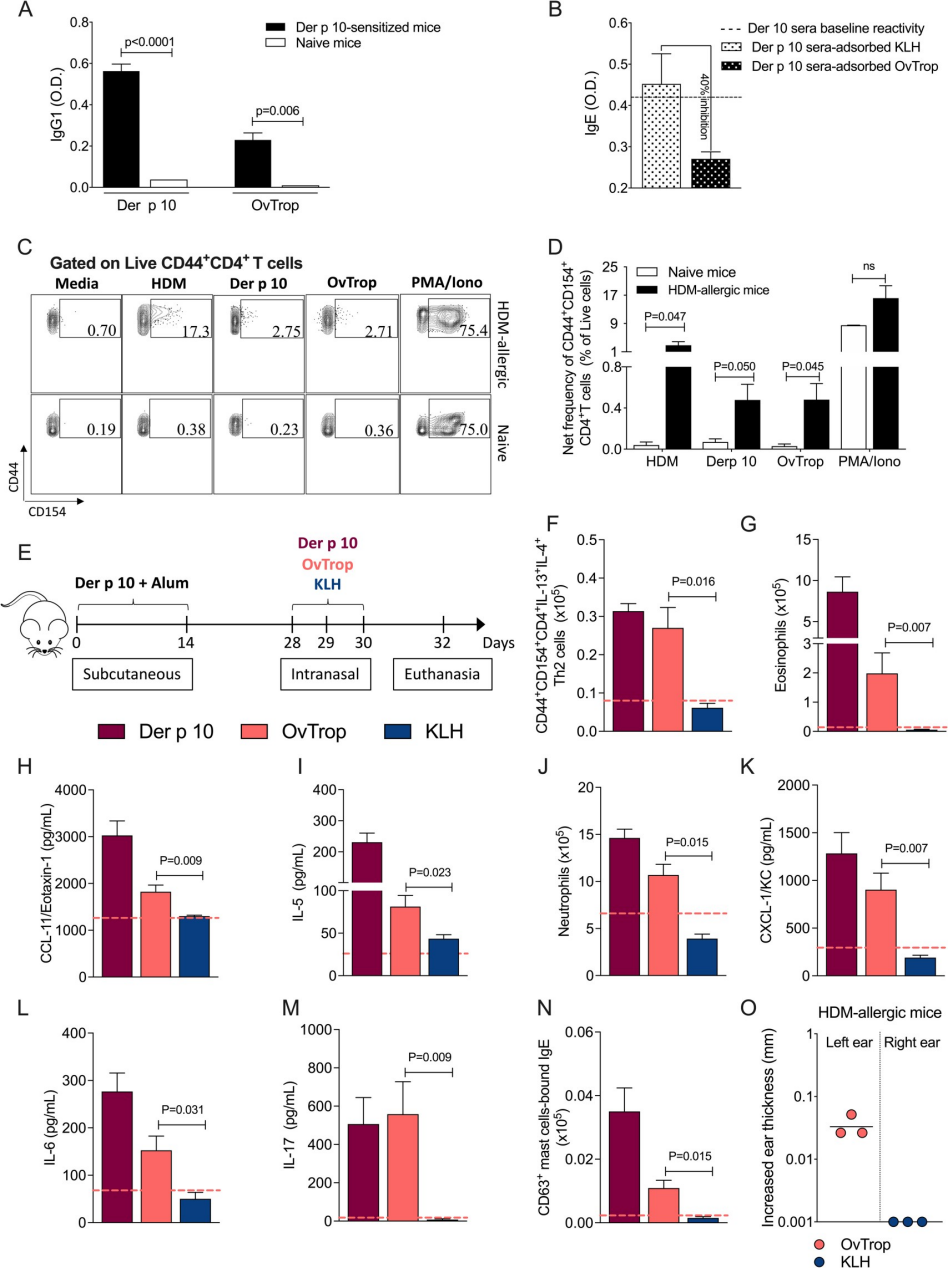
Homologous helminth proteins exacerbate pulmonary type-2 allergic inflammation in house dust mite presensitized mice. (A) Der p 10- and OvTrop-specific IgG1 levels in serum pools from naïve and Der p 10-sensitized mice. (B) Competitive ELISA assay for Der p 10-specific IgE reactivity using OvTrop-adsorbed Der p 10-sera, a non-related protein (KLH)-adsorbed Der p 10-sera, as well as a non-adsorbed Der p 10-sera (dotted line). (C) In vivo cross-sensitization model to test the capacity of the helminth tropomyosin (OvTrop) to elicit a pulmonary type-2 allergic inflammation in Der p 10-systemically presensitized mice. Balb/c mice were sensitized by subcutaneously with rDer p 10-alum on days 0 and 14. On days 28–30, mice were challenged intranasally with either rDer p 10, rOvTrop or KLH. Non-sensitized mice but challenged with the proteins were used as controls (pink dotted line for OvTrop). (D-M) The characterization of the lung inflammation by immunophenotypic flow cytometry analysis of memory antigen-specific Th2 effector cells, Siglec-F^+^ eosinophils, Ly6G^+^ neutrophils, and by profiling CCL-11 (Eotaxin-1), CXCL-1 (KC), IL-5, IL-6 and IL-17 in lung tissue homogenate. (N) Another flow cytometry analysis was addressed specifically to quantify the frequency of FceR1^+^c-Kit^+^ mast cells bound-IgE, expressing CD63 as an activation/degranulation marker. (O) Skin test reactivity in HDM-allergic mice following intradermal injection of helminth-derived recombinant tropomyosin (filled diamonds) or KLH (open diamonds) Bars represent the mean + SEM and geometric means were used for ear thickness. P values are indicated on each graph.

To assess the impact of the cross-reactivity between HDM- and *Ascaris*-tropomyosin in the allergic effector response, we used a highly homologous filarial tropomyosin (see S3 Fig) and Der p 10 in pulmonary sensitization experiments. To this end, IgG1 from sera from mice sensitized with rDer p 10 in alum reacted quite similarly to either Der p 10 or to its helminth homologue, OvTrop (Fig 7A). Partial cross-reactivity at the level of IgE could also be shown using a competitive ELISA assay in which OvTrop-adsorbed sera from Der p 10-sensitized mice showed a 40% reduction in IgE reactivity to Der p-10 when compared to sera adsorbed with the non-related protein (KLH) or to unadsorbed sera from Der p 10 sensitized mice (Fig 7B).

At the level of the T cell, we were also able to show the importance of the cross-reactive antigens. As can be seen in Fig 7C and 7D, a subset of memory antigen-activated CD4^+^ T cells (expressing both CD44 and CD154 [CD40L]) in the lungs of HDM-sensitized mice expand in marked frequencies after re-stimulation with HDM extract. Indeed, both Der p 10 and OvTrop also induced a significant increase in the CD4^+^CD44^+^CD40L^+^ T cells when compared to comparing with the appropriate controls (unstimulated cells and naïve mice lung cells).

In addition, OvTrop was able to substitute for Der p 10 in driving pulmonary type-2 allergic inflammation in Der p 10-presensitized mice (Fig 7E–7N). Overall, allergic sensitization with Der p 10 followed by the intranasal challenge with Der p 10 elicited a type-2 enriched environment, with elevated frequencies of memory antigen-activated IL-4 and IL-13 producing Th2 cells, eosinophils, neutrophils, and a significant increase of Th2 and Th17-related cytokines and chemokines. Moreover, challenge with Der p 10 was also able to increase the frequency of activated lung mast cells bound-IgE expressing CD63, a marker for degranulation. Interestingly, challenge with the cross-reactive OvTrop resulted in a significant increase in the memory IL-4 and IL-13 producing Th2 cells (0.26 ± 0.05 x10^5^ cells) in the lungs of Der p 10-presensitized mice, an expansion that did not occur when challenged with a control antigen, KLH (0.06 ± 0.01 x10^5^ cells, P = 0.016) (Fig 7F). Furthermore, OvTrop also induced an eosinophil-rich inflammation characterized by elevated frequencies of Siglec-F^+^ eosinophils (P = 0.007), and high levels of CCL-11 (eotaxin-1) (P = 0.009) and IL-5 (P = 0.023) (Fig 7G–7I). The inflammatory response in the lungs driven by OvTrop, Der p 10, or by HDM extract (from previous observations) also induced a type 17-like inflammatory profile with significantly increased levels of neutrophils, CXCL-1, IL-6 and IL-17 (Fig 7J–7M). Finally, OvTrop promoted the expansion of activated mast cell bound-IgE that contrasted with that seen in cells stimulated with KLH (0.010±0.005 x10^5^ cells vs 0.001 ± 0.0008 x10^5^ cells, P = 0.015) (Fig 7N). This set of data suggests that the cross-reactivity between HDM and helminths antigens (e.g., Der p 10 and OvTrop) may exacerbate the mixed Th2/Th17 pulmonary allergic inflammation.

Finally, to examine this concept of cross-sensitization *in vivo*, we tested the capacity of OvTrop to cross-link HDM-specific IgE antibodies using intradermal skin testing with the ears of mice of HDM-sensitized mice. As seen, helminth tropomyosin injection in the ear of HDM-allergic mice induced an almost twenty-fold increase in ear thickness when compared to that seen by KLH (Fig 7O).

### HDM-driven cross-reactive antibodies do not mediate *Ascaris* larval killing

**Fig 8:**
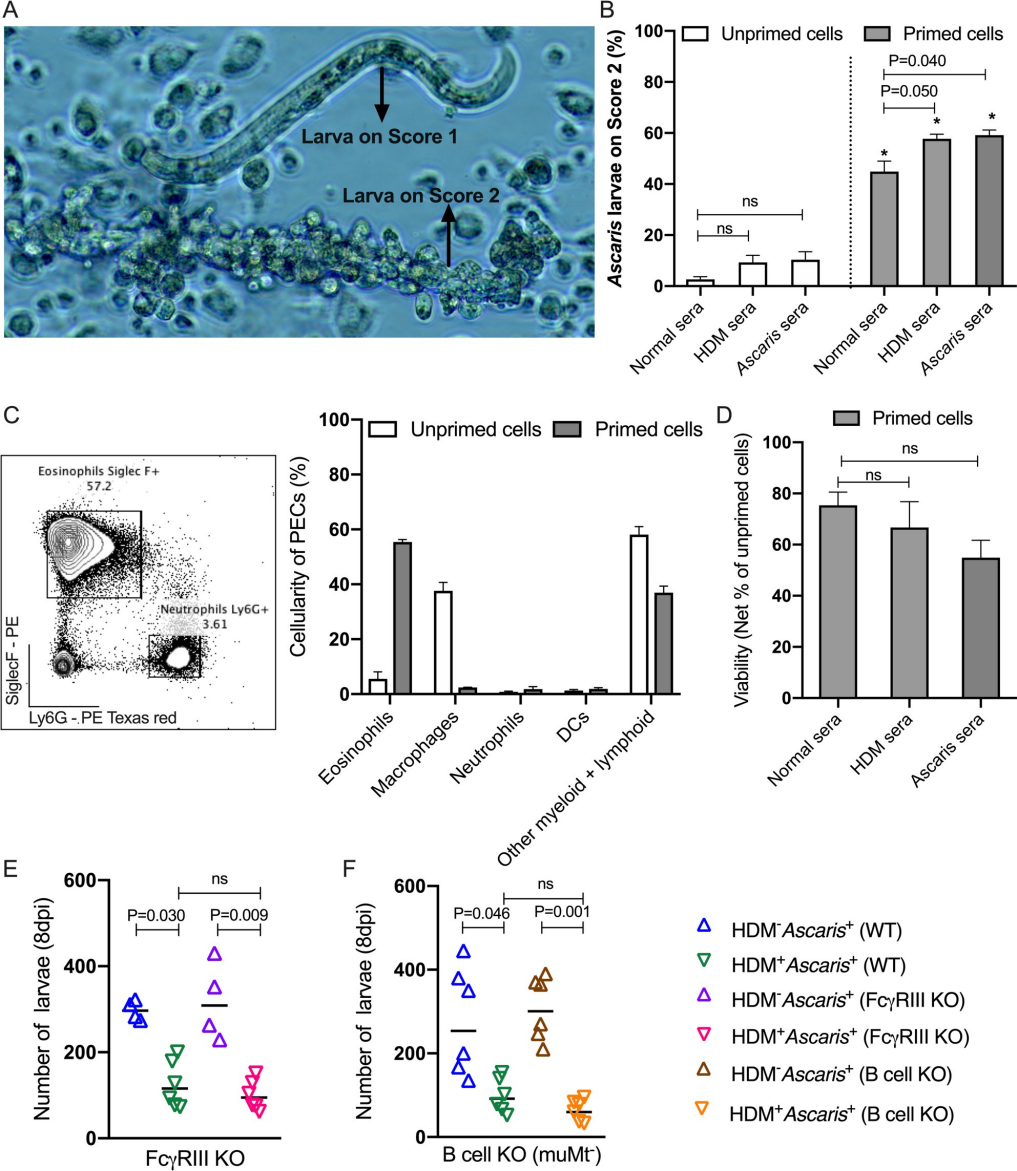
Antibodies may facilitate PEC adherence to *Ascaris* larvae but HDM-driven cross-reacted antibodies do not mediate larval killing. Larvae-associated peritoneal cavity cells (PECs) adherent the surface was assessed by microscopy and scored (1 for few to no adherent PECs and 2 for adherent PECs (A). Percentage of larvae with unprimed and primed PECs on their surface (Score 2) in the presence of the different sera pools (B). Representative flow cytometry dot plot for primed PECs and the frequency of the immunophenotyped cells (C). Larval viability measured by motility (D). Parasite burden in the lungs HDM^–^Ascaris^+^ and HDM^+^Ascaris^+^ WT C57/BL6 mice with HDM^–^Ascaris^+^ and HDM^+^Ascaris^+^ FcγRIII-deficient (E) and HDM^–^Ascaris^+^ and HDM^+^*Ascaris*^+^ muMt mice (F).

To understand the role played by antibodies in mediating cellular attachment or killing of *Ascaris* larvae, we incubated larvae with unprimed peritoneal cells from naïve mice or primed peritoneal cells, in the presence or absence of HDM-sensitized sera or sera from *Ascaris*-infected mice (Fig 8). Peritoneal cells from naïve mice were incapable of attaching to *Ascaris* larvae even in the presence of sera from sensitized mice (Fig 8B). Moreover, the viability of *Ascaris* larvae incubated with unprimed cells in all conditions was 100% after 24 hours in culture. Using primed peritoneal cells, that were composed largely of activated eosinophils showed marked adherence to the larval surface, even in the presence of sera from naïve mice. Moreover, there was a significant increase in the number of cells attached to the larvae in the presence of HDM-allergic sera or *Ascaris-*immune sera (Fig 8B). These data suggest that activated cells, by themselves, are able to attach to the surface of helminth larvae with some increase in binding when antibodies are present. Although the viability of the helminth larvae was reduced when activated cells were used (when compared to cells from naïve mice) the cell-mediated larval killing appeared to be independent of HDM-specific antibodies (Fig 8D). Moreover, the number of *Ascaris* larvae that migrate to the lungs of HDM-sensitized mice deficient either in B cells [(Fig 8E) confirmed by immunophenotypic analysis (S5 Fig)] or deficient in the alpha-chain of FcγRIII (Fig 8F) revealed that the HDM-allergen driven cell-mediated larval killing occurs independent of the presence of antibodies.

## Discussion

Helminth parasites belong to a diverse group of complex metazoans from different taxonomic families, showing notable differences in their biological life cycles along with marked variation in tissue tropism [18]. These differences may drive different clinical outcomes, but they may also help to explain the contrasting results seen among studies examining their role of atopy in modulating or enhancing allergic inflammation. These contrasting studies commonly fail to take into consideration, the species of the helminth, the tissue affected, the intensity of the helminth infection, or the nature of the allergic disease assessed. Each of these factors may have fundamental roles in driving the outcomes seen.

Helminth parasite antigens and allergens, in general, are well known for having common properties as they both trigger an IgE-dominated type-2 response [5,19–23]. It has been suggested that there is a high degree of molecular and structural similarities among helminth antigens with many common allergens [24]. Indeed, the high degree of homology of certain B-cell epitopes between helminth parasites and allergens have been implicated in the pathogenesis and/or regulation of both helminth infection and allergic disorders [12–14]. Several molecules from common aeroallergens, including those from *D*. *pteronyssinus* (Der p 1, Der p 8 and Der p 10), *Blomia tropicalis* (Blo t 8) proteins, and *Blattella germanica* (Bla g 5 proteins) have been characterized as having significant IgE cross-reactivity with helminth proteins from a variety of parasites including *Ascaris* and the filarial parasites [25–27].

Using a murine model of HDM-induced allergic pulmonary inflammation followed by *Ascaris* infection we were able to show that HDM sensitization, by itself, can drive IgE and IgG1 antibodies that specifically recognize surface-expressed proteins on *Ascaris* larvae, and have identified these *Ascaris*-encoded proteins as tropomyosin and enolase. Tropomyosins belong to a family of highly conserved proteins with multiple isoforms present in both muscle and non-muscle cells [28]. Nonhuman vertebrate tropomyosins are not typically allergenic to humans whereas tropomyosins from invertebrates (e.g. shrimp, dust mite, cockroach, helminth nematodes, *A*. *aegypti* mosquito) are considered major allergens [26,29–31]. Mite tropomyosins are classified as group 10 allergens (e.g. Der p 10, Der f 10, Blo t 10, Lep d 10, Tyr p 10) whereas they are characterized differently in cockroach (group 7) and in nematodes (Group 3) including *Ascaris* (Asc l 3) [23]. It has been shown that filarial parasite *Onchocerca volvulus* tropomyosin (OvTrop) and HDM tropomyosin (Der p 10) are very conserved with 72% similarity at the amino acid level, a degree of homology that has already been shown to drive a strong cross-reactive antibody response [26]. In the present study, we have shown in well-controlled *in vivo* experiments that HDM sensitization induces a strong cross-reactive antibody response to *Ascaris* tropomyosin. This may provide an explanation for the high frequency of asthma and other allergic diseases detected in certain tropical settings. In fact, in a meta-analysis of 30 clinical studies, *Ascaris lumbricoides* infection was associated with aggravated asthma symptoms. [32]. Given the high degree of molecular similarity between *Ascaris* and *Onchocerca volvulus* (95%), it could be expected that IgE-binding epitopes in Der p 10 and OvTrop may be similar to those observed in *Ascaris* tropomyosin. Moreover, some other studies have reported cross-reactivity between *Ascaris* and cockroach tropomyosins (Asc l 3 and Bla g 7, respectively) [25] or with *Blomia tropicalis* (Blo t 10) [33]. Indeed, IgE epitope mapping prediction [34] revealed that *Ascaris* tropomyosin has 10 epitopes highly conserved for IgE binding along its full length-amino acid sequence, highlighting its allergenicity.

When we investigated the biological function of the cross-reactivity induced by the allergens (Der p 10) and helminth (OvTrop) tropomyosins in the context of the allergic inflammation effector responses, we demonstrated that the challenge in the lungs with OvTrop in Der p 10-systemically presensitized mice resulted in a significant influx of inflammatory cells, including a subset of memory IL-4 and/or IL-13 producing Th2 cells, associated with a robust eosinophil-rich type-2 inflammation (IL-5 and eotaxin-1) and elevated numbers of activated IgE-sensitized mast cells in the lungs. These data indicate that, upon presensitization with allergens, helminth proteins can exacerbate the systemic Th2 response as well as type-2 inflammation in the tissue, both of which are strongly associated with the pathogenesis of the allergic diseases.

Enolases have also been described as major allergens with homologous IgE-binding epitopes described in different fungal species [35–37] as well as in *Hevea* late*x* (Hev b 9) [38]. However, epitope mapping scans on the *Ascaris* enolase protein failed to show potential IgE-binding sites, which may explain why there was little to no IgE (see Fig 4E). *Ascaris suum* enolase (As-enol-1) has been extensively studied over the last few years as potential vaccine candidate and [39] in fact, RNA interference of the *A*. *suum* enolase mRNA led to significantly shorter larvae, suggesting that loss of enolase expression may impair larval development [40,41].

Although there is little consensus within the scientific community, it has been demonstrated that eosinophils can mediate resistance to certain helminth infections [42–47] based on studies in humans and in experimental models. However, how these eosinophils kill the helminths remains elusive. Rainbird et al. [46] suggested that the eosinophils kill helminth parasites based on their activation status [48] possibly through a serum-dependent system (either complement- or antibody- associated with activation of complement system by the alternative pathway [43]), or through an antibody-dependent mechanism [17,46]. Our *in vivo* studies in allergic mice deficient either in B cells or in the alpha-chain FcγRIII reveals, however, that the HDM-allergen driven cell-mediated larval arrest and killing is not antibody-dependent (see Fig 7E and 7F).

In summary, our data suggest that HDM allergen sensitization drives *Ascaris* and other helminth reactive antibodies through the molecular and structural similarity among homologous tropomyosins and enolases, which may have direct implications in the outcome of allergic inflammation and provides an additional layer of complexity to the helminth-allergy debate.

## Methods

### Ethics statement

The maintenance and use of animals for this study were approved by NIH Animal Care and Use Committee (Protocol #LPD-6).

### Mice

BALB/c mice (male, 8-weeks old) (Taconic Farm), C57BL/6J (male, 8-weeks old) (Jackson), FcγRIII knock-out (KO) mice (male, 8-weeks old) (B6.129P2-Fcgr3tm1Jsv/J; Jackson) and B cell muMt mice (male, 8-weeks old) (B6.129S2-Ighmtm1Cgn/J, Jackson) were used throughout the study.

### Allergic sensitization

Mice were sensitized intranasally with 200μg in 30μL of HDM extract (Greer Laboratories) at day 0 and day 7. At day 14, 16, and 18, mice were given 50μg in 30μL of HDM extract. Mice were infected with *Ascaris* at day 18, immediately after the last sensitization with HDM. Antibody responses were examined at day 23 (5 days post infection [dpi]), day 26 (8 dpi) and day 36 (18 dpi). Chronic allergic sensitization was induced by intranasal sensitization with 200μg in 30μL of HDM extract at day 0. At days 7 and 14, 100μg in 30μL of HDM extract. From day 21 (week 3), mice were given 50μg in 30μL of HDM extract twice a week for a period of 5 weeks.

The *in vivo* cross-sensitization was assessed by the capacity of OvTrop to elicit a pulmonary type-2 allergic inflammatory response in Der p 10-systemically primed mice. Balb/c mice were systemically sensitized by subcutaneous injections of 20 μg/mL of rDer p 10-alum on days 0 and 14. On days 28–30, mice were challenged intranasally with either rDer p 10 (40 μg), rOvTrop (40μg) or KLH (40μg). rDer p 10 and KLH (unrelated protein) were used as positive and negative controls of the assay. Non-sensitized mice but challenged with the proteins were used as controls.

### Parasites

*Ascaris suum* eggs were isolated from female uteri through gentle mechanical maceration, purified by straining, and cultured to embryonation in 0.2 M H_2_SO_4_ as described by Boes et al. [49]. On day 100 of culture, the peak of larvae infectivity [50], the fully embryonated eggs were used for experimental infections.

### Parasitological analysis and larval recovery and *Ascaris*-immune serum

Mice were inoculated intragastrically with 2,500 fully embryonated eggs. Serum samples were collected from all animals at different time points before euthanasia. Parasite burden was evaluated as previously described [50]. To generate an *Ascaris*-immune serum pool, 3 rounds of infections with 2,500 fully embryonated eggs were performed as described [51]. The animals were bled, and the sera was pooled fourteen days after the final infection.

### *Ascaris* lung-stage larval antigen preparation

The larvae recovered from the lungs and airways of wild type (WT) BALB/c infected mice at day 8 post-infection were purified and used to prepare antigen as described previously [17].

### Der p 10 and helminth tropomyosin

Baculovirus expressed tropomyosins from *Dermatophagoides pteronyssinus* (Der p 10) or from *Onchocerca volvulus* (OvTrop) [26] were used. Commercially available recombinant Der p 10 (rDer p 10) (Indoor biotechnologies) were also used.

### Antibody response

IgG1, IgG2, IgG3, and IgE responses to HDM extract, *Ascaris* antigen, Der p 10 and OvTrop were evaluated by ELISA in which Immulon 4HBX plates were coated with 5μg/well of HDM extract (Greer Laboratories), 5ug/well of rDer p 10 or 5μg/well of OvTrop using standard techniques [16].

### Confocal imaging of *Ascaris* larval stages

Infective L3, hatched from fully embryonated eggs [17], and lung-stage larvae recovered from the airways of infected-mice at 8 dpi, were incubated for 1 hour at RT, with 2 drops/mL of Hoechst 33342 (Invitrogen, USA). After incubation, the larvae were washed and incubated at 4°C overnight in 1:10 dilutions (1x PBS) of negative sera from naïve mice; allergic sera from HDM-sensitized mice; or *Ascaris*-immune sera. After the incubation and washes, the larvae were incubated with Alexa-Fluor 594-conjugated anti-mouse IgG1 (Invitrogen, USA) diluted 1:500 in 1% BSA-PBS. For the evaluation of IgE-binding, the L3 larvae were incubated at 4°C overnight with IgG-depleted undiluted naïve sera, HDM-allergic sera and *Ascaris*-immune sera. After the incubation and washes, the larvae were incubated with biotinylated rat anti-mouse IgE (Invitrogen, USA) diluted 1:100 in 1% BSA-PBS, followed by an incubation with streptavidin-PE diluted 1:50 in 1% BSA-PBS. All stained larvae were imaged using Leica SP8 inverted confocal microscope (Leica Microsystems). The microscope was equipped with full range of visible lasers, internal ultra-sensitive hybrid detectors (HyDs), a motorized stage, and Lx20.0 multi-immersion objective with 700 um working distance. Diode laser was used for 405 nm excitation; Argon laser for 488 nm excitation; and DPSS laser for 594 nm excitation wavelengths. Detection gates were set as follows: channel one 415–460 nm; channel two 498–530 nm; channel three 604–630 nm. Tiled images of whole larvae were acquired using Tilescan application of LAS X. Z stacks consisting of 6–8 single planes (5 μm each over a total tissue depth of 40 μm) were acquired. Post-acquisition analysis was performed using Imaris software (Imaris version 9.2.1, Bitplane AG, Zurich, Switzerland).

### 2D electrophoresis and immunoblotting of *Ascaris* larvae proteins

Two-dimensional gel electrophoresis was performed on 200 μg of Ascaris lung-stage larval antigens using standard techniques by Kendrick Labs, Inc. (Madison, WI). Briefly, two-dimensional electrophoresis was performed according to the carrier ampholine method of isoelectric focusing [52,53]. SDS slab gel electrophoresis was carried out for about 4 hrs at 15 mA/gel. The following proteins (Millipore Sigma) were used as molecular weight standards: myosin (220,000), phosphorylase A (94,000), catalase (60,000), actin (43,000), carbonic anhydrase (29,000), and lysozyme (14,000). These standards appear along the basic edge of the Coomassie blue-stained 10% acrylamide slab gel. The gel was dried between sheets of cellophane with the acid edge to the left. After slab gel electrophoresis, the duplicate gel for blotting was placed in transfer buffer (10mMCaps, pH 11.0, 10% MeoH) and transblotted onto a PVDF membrane overnight at 200 mA and approximately 100 volts/ two gels. The PVDF membrane was wet in 100% methanol, rinsed briefly in tween-20 tris buffer saline (TTBS), and blocked for two hours in 5% bovine serum albumin (BSA) diluted in TTBS. The blot was then incubated in primary antibody (HDM-specific sera) diluted 1:5,000 in 2% BSA TTBS) overnight and rinsed 3 x 10 minutes in TTBS. The blots were placed in secondary antibody anti-mouse IgG-HRP, 1: 2,000 diluted in 2% BSA TTBS for two hours, rinsed as above, treated with ECL, and exposed to x-ray film.

### Protein digestion and peptide extraction

Identified proteins from the 2D electrophoresis were excised, washed, and trypsin digested based on published protocols [54–56]. Small amounts of human keratin were use as positive control for the digestion efficiency of trypsin in the assay, explaining the presence of this human protein in sample D.

### Nano LC-MS/MS

The peptide mixtures were analyzed by reversed phase nanoliquid chromatography (LC) and MS (LC-MS/MS) using a NanoAcuity UPLC (Micromass/Waters, Milford, MA) coupled to a Q-TOF Xevo G2 mass spectrometer (Micromass/Waters, Milford, MA), according to published procedures [54,57–59]

### Data processing and protein identification

The raw data were processed using ProteinLynx Global Server (PLGS, version 2.4) software as previously described [59]. The resulting pkl files were submitted for database search and protein identification using an in-house Mascot server (www.matrixscience.com, Matrix Science, London, UK) and against an in-house PLGS database version 2.4 (www.waters.com). The Mascot and PLGS database search provided a list of proteins for each gel band. To eliminate false positive results, for the proteins identified by either one peptide or a mascot scores lower than 25, we verified the MS/MS spectra that led to identification of a protein.

### *Ascaris* and HDM homolog proteins structures

The identified antigenic targets sequences for *Ascaris lumbricoides* tropomyosin (GenBank ACN32322.1), *Ascaris suum* enolase (GenBank ADQ00605.1) and *Ascaris suum* putative enoyl-CoA hydratase (ERG87856.1) were selected for molecular and structural similarity analysis. *D*. *pteronyssinus* tropomyosin, also known as Der p 10 (NCBI Reference Sequence XP_027200893), *D*. *pteronyssinus* enolase-like (NCBI Reference Sequence: XP_027197319.1) and *D*. *pteronyssinus* enoyl-CoA hydratase, and mitochondrial-like (NCBI Reference Sequence: XP_027198819.1) sequences were aligned with *Ascaris* sequences in MegAlign (version 11.0.0; DNASTAR), using ClustalW. For 3D structure prediction and modeling, the protein sequences were submitted to the homology modeling server I-TASSER 3.0 (available at: http://zhanglab.ccmb.med.umich.edu/I-TASSER) for protein structure prediction. The top models in terms of C-score were analyzed using UCSF Chimera (v.1.14, University of California) for structure comparison.

### Phylogenetic analysis of enolase and tropomyosin

Alignments of tropomyosin, enolase and enoyl-CoA-hydratase with their helminth orthologues (www.parasite.wormbase.org) and/or known allergens (COMPARE ALLERGEN DATABASE, https://comparedatabase.org) were generated using the muscle algorithm in MegAlignPro (Lasergene DNASTAR v 17.0.2). The aligned fasta files were used as input to RAxML (v 7.8.6) and the trees were computed using GAMMA model (-m PROTGAMMAAUTO) with rapid bootstrap of 1000 (-# 1000). The resulting trees were visualized in FigTree v 1.4.4.

### Luciferase Immunoprecipitation System (LIPS) Assay

Fusion proteins of the *Ascaris* tropomyosin, enolase and enoyl-CoA hydratase, were generated by cloning the full-length coding sequence of the proteins into the mammalian *Renilla reniformis* luciferase (Ruc)-containing expression vector pREN2, and transfecting 293F cells (Thermo Fisher Scientific) as previously described [60]. The IgG and IgE responses to the three fusion proteins were measured using LIPS antibody assays [61,62]. Output was measured as relative light units (RLU) using a Berthold LB 960 Centro microplate luminometer and coelenterazine substrate mixture (Promega, Madison WI).

### Competitive ELISA for Der p 10-specific IgE responses

Immulon 4 plates was coated with OvTrop (10 μg/well) or (KLH) (10 μg/well). Another set of plate for measuring the Der p 10-specific IgE response was coated with Der p 10 (5 μg/well). Both sets were incubated overnight at 4C, then blocked with 200 μl of blocking buffer (PBS 1X, 0.05% Tween-20 containing 5% BSA). The IgE detection ELISA plate was placed at 4C until later use. Three pools of 50 μl of undiluted IgG-depleted (using Pierce Protein A/G Ultralink Resin (ThermoFisher, Waltham, USA) sera was added to both OvTrop and KLH antigen-containing wells and incubated overnight at 4°C. 50uL of each pool were transferred to the Der p 10-coated plate, following incubation for 1 hour at RT. After washing, 50 μl of 1:250 (diluted in PBS 1X, 0.05% Tween-20 containing 1% BSA) biotinylated anti-mouse IgE (Invitrogen, Carlsbad, USA) were added to the appropriate wells and incubated 1 hour at RT. Following an additional wash step, 50μl of streptavidin-conjugated to HRP (R&D Systems, Minneapolis, USA) was added diluted 1:100 and incubated for 30 minutes at RT. A final wash step was performed and 50μl of 1Step Ultra TMB-ELISA Substrate (ThermoFisher) was added to each well and the colorimetric reaction was stopped after 10 minutes 30 minutes by adding 25 μl of H_2_SO_4_. The plate was then read at 450 nm.

### Homologous proteins cross-reactivity at T cell level

The cross-reactivity between HDM and helminth homologue proteins was also assessed at T cell level by stimulating the inflammatory lung cells from mice chronically sensitized with house dust mite extract, with different conditions. Briefly, lung cells from HDM-allergic mice were obtained as described by [16] were incubated overnight at 37°C, 5% CO2, in the absence (media) or upon stimulation with HDM extract (10μg/mL), rDer p 10 (10μg/mL), OvTrop (10μg/mL) and finally 0.5/0.05 nM of PMA/ionomycin (P/I) (Sigma-Aldrich, USA). 10ug/mL of Brefeldin A was added in all conditions. Cells were then stained using a panel of antibodies to profile the antigen-specific effector memory CD4^+^ T cells expressing the activation marker CD40L.

Briefly, cells were stained with a Live/Dead marker (Fixable Blue-UV450). Secondly, after washing with FACS buffer (PBS, 2% FBS), antibodies to the extracellular markers CD19, TCR-b, CD44 were used. After washing, cells were then fixed with 4.2% formaldehyde (BD Cytofix, BD Biosciences) and permeabilized (Perm buffer eBiosciences), and finally stained with CD4, CD40L antibodies (flow cytometry panel in S1 Table).

### *In vivo* cross-sensitization

The characterization of the lung inflammation elicited by OvTrop in Der p-10 sensitized mice was assessed by profiling the cytokine milieu in the tissue homogenate by Luminex (Millipore, USA), including CCL-11 (Eotaxin-1), CXCL-1 (KC), IL-4, IL-5, IL-6 and IL-17A, and by immunophenotyping the inflammatory cells with a specific interest on the populations of Siglec-F^+^ eosinophils, Ly6G^+^ neutrophils and FceR1^+^c-Kit^+^ mast cells bound-IgE, expressing CD63 as a degranulation marker (S4 Fig and flow cytometry panel in S2 Table).

Moreover, the *in vivo* cross-reactivity was also assessed by skin test reactivity using an ear swelling model with some modifications [27]. Briefly, HDM-presensitized mice received intradermal injections with 10μL (2mg/mL) of OvTrop (left ear) or 10μL of KLH (Keyhole limpet hemocyanin—a non-related antigen) (2mg/mL) (right ear). Ear thickness was measured with a caliper before and 60 minutes after all injections. Skin reactivity was represented by the increase of the ear thickness after the injection over the baseline.

### *In vitro* assay for cell association on larvae surface and larval viability

To obtain primed inflammatory cells, BALB/c WT mice were inoculated intraperitoneally with 10,000 fully embryonated eggs to induce a local eosinophilia, as demonstrated previously [63]. Ten days post-injection, the peritoneal cavity cells were collected and immunophenotyped by flow cytometry (panel in S3 Table). Unprimed cells were collected from peritoneal cavity of naïve BALB/c WT mice. For the *in vitro* assay, 2.5 x 10^5^ primed or unprimed cells were co-cultured with 250 *Ascaris* L3 larvae. Heat-inactivated sera from naïve, HDM-allergic and *Ascaris*-immune mice, were pooled separately and added at 1:10 dilution in triplicate wells and incubated at 37°C in 5% CO_2_ for 24 hours. The cells associated with the surface of the larvae were assessed 2 hours after incubation, using an inverted microscope coupled to a digital camera and scored. Score 1 indicates no cells adhered to the larvae surface and score 2 indicates cells attached to the larvae surface. Larval viability was measured by motility 24 hours after incubation.

### Statistical analyses

Unless stated otherwise, geometric means (GM) and 95% confidence intervals (CI) were used as the measure of central tendency. For non-parametric data, the Kruskal-Wallis test followed by Dunn’s Multiple Comparison Test was used for the analysis of HDM-specific and *Ascaris*-specific antibody responses. The Mann-Whitney U test was used to determine the differences in the parasite burden between HDM^-^Ascaris^+^ and HDM^+^Ascaris^+^ mice and antibody reactivity by LIPS assay. Statistical analysis was performed using Prism 8.0 (GraphPad Software Inc., USA), and the differences were considered statistically significant when P< 0.05.

## Supporting information

S1 Fig HDM- and *Ascaris*-specific IgE and IgG1 levels using sera from acute and chronic allergic mice. HDM-specific IgE and IgG1 (A-B) and *Ascaris*-specific IgE and IgG1 (C-D) levels in non-allergic and non-infected naive mice; HDM-sensitized allergic mice; and chronically HDM-sensitized mice. P values are indicated on each graph. and * indicates significantly different (p<0.05) from naïve group.
(TIFF)

S2 Fig IgE binding in the surface of early *Ascaris* migrating larval-stages in the presence of normal, HDM-allergic and Ascaris-immune sera. Representative confocal microscopy images highlighting the IgE (red) staining in the surface of infective L3 larvae using sera from normal mice (A), HDM-sensitized mice (B), and *Ascaris*-immune sera (C). Autofluorescence (green) is also seen.
(TIFF)

S3 Fig Protein sequences and structural similarities of tropomyosin among helminths and allergens. Alignment of *Onchocerca volvulus*, *Ascaris* and HDM tropomyosin (Der p 10) sequences showing identical (shaded in black) amino acids (A) and the predicted 2D structure of *Onchocerca volvulus (beige)* and *Ascaris* tropomyosin (red) for comparison (B).
(TIFF)

S4 Fig Gating strategy for the myeloid compartment in the lungs of the mice including Siglec-F^+^ eosinophils, Ly6G^+^ neutrophils, and c-Kit^+^FceRIa^+^IgE^+^ mast cells. (TIFF)

S5 Fig Gating strategy for CD19^+^B220^+^ B cell frequency in the lungs of HDM-allergic WT mice (above) and HDM-allergic uMT mice (below). (TIFF)

S1 Table Flow cytometry antibodies. (DOCX)

S2 Table Flow cytometry antibodies. (DOCX)

S3 Table Flow cytometry antibodies. (DOCX)
