# Supplementary figures and images for: House dust mite sensitization drives cross-reactive immune responses to homologous helminth proteins
Source: PLoS Pathog. 2021 Mar 2;17(3):e1009337. doi: 10.1371/journal.ppat.1009337 (PMC7924806; doi:10.1371/journal.ppat.1009337)

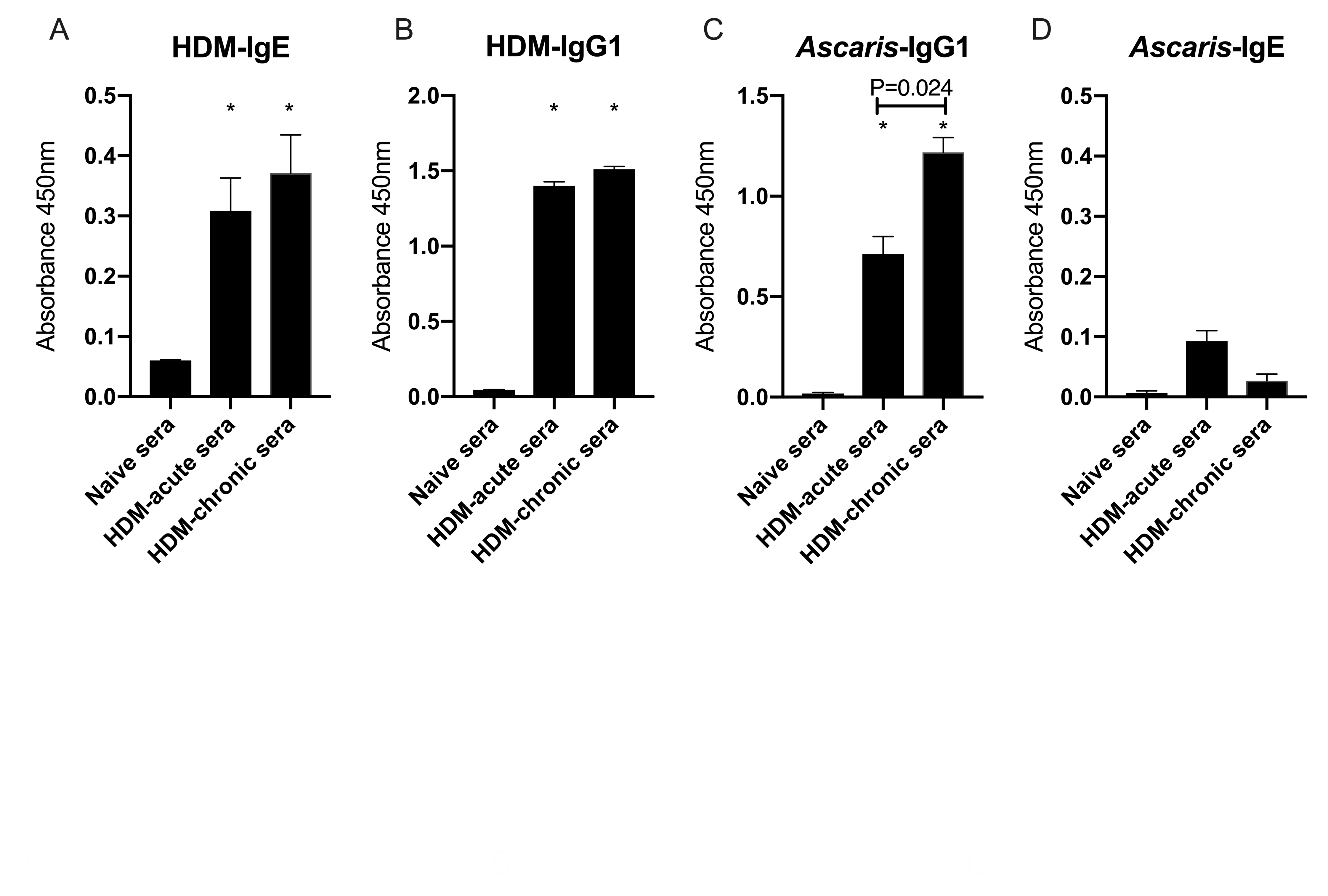

Supplement: S1 Fig — HDM-specific IgE and IgG1 (A-B) and Ascaris-specific IgE and IgG1 (C-D) levels in non-allergic and non-infected naive mice; HDM-sensitized allergic mice; and chronically HDM-sensitized mice. P values are indicated on each graph. and * indicates significantly different (p<0.05) from naïve group. (TIFF) [file ppat.1009337.s001.tiff]

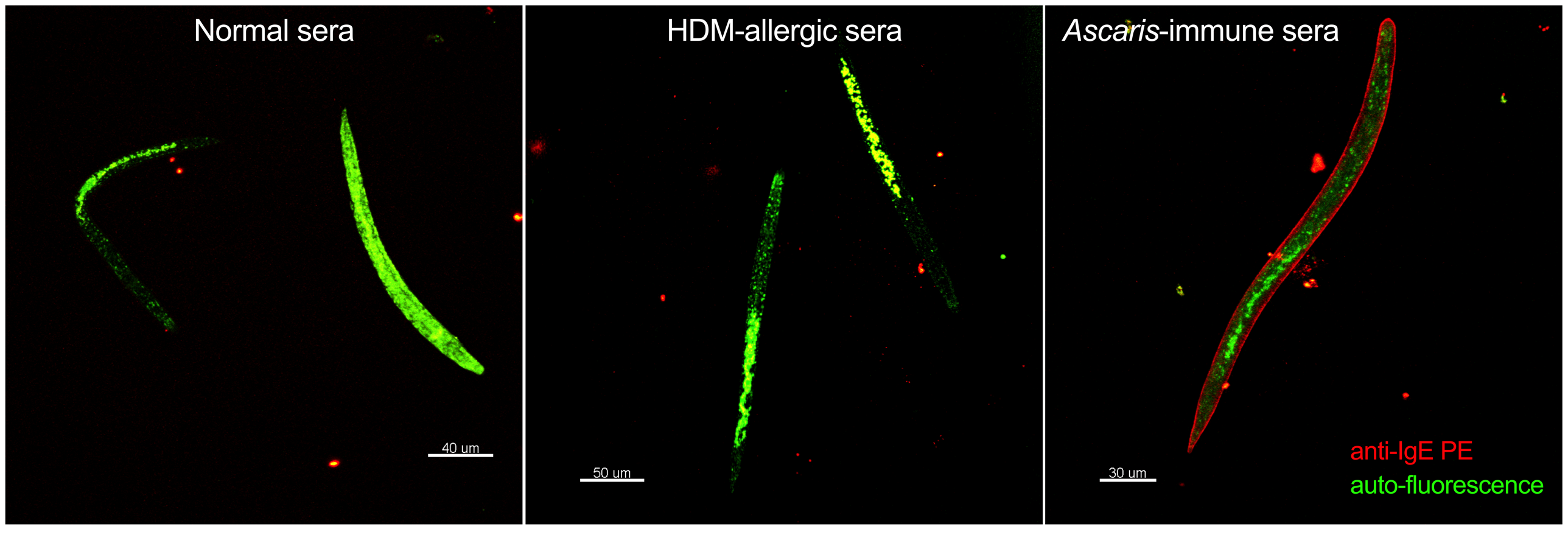

Supplement: S2 Fig — Representative confocal microscopy images highlighting the IgE (red) staining in the surface of infective L3 larvae using sera from normal mice (A), HDM-sensitized mice (B), and Ascaris-immune sera (C). Autofluorescence (green) is also seen. (TIFF) [file ppat.1009337.s002.tiff]

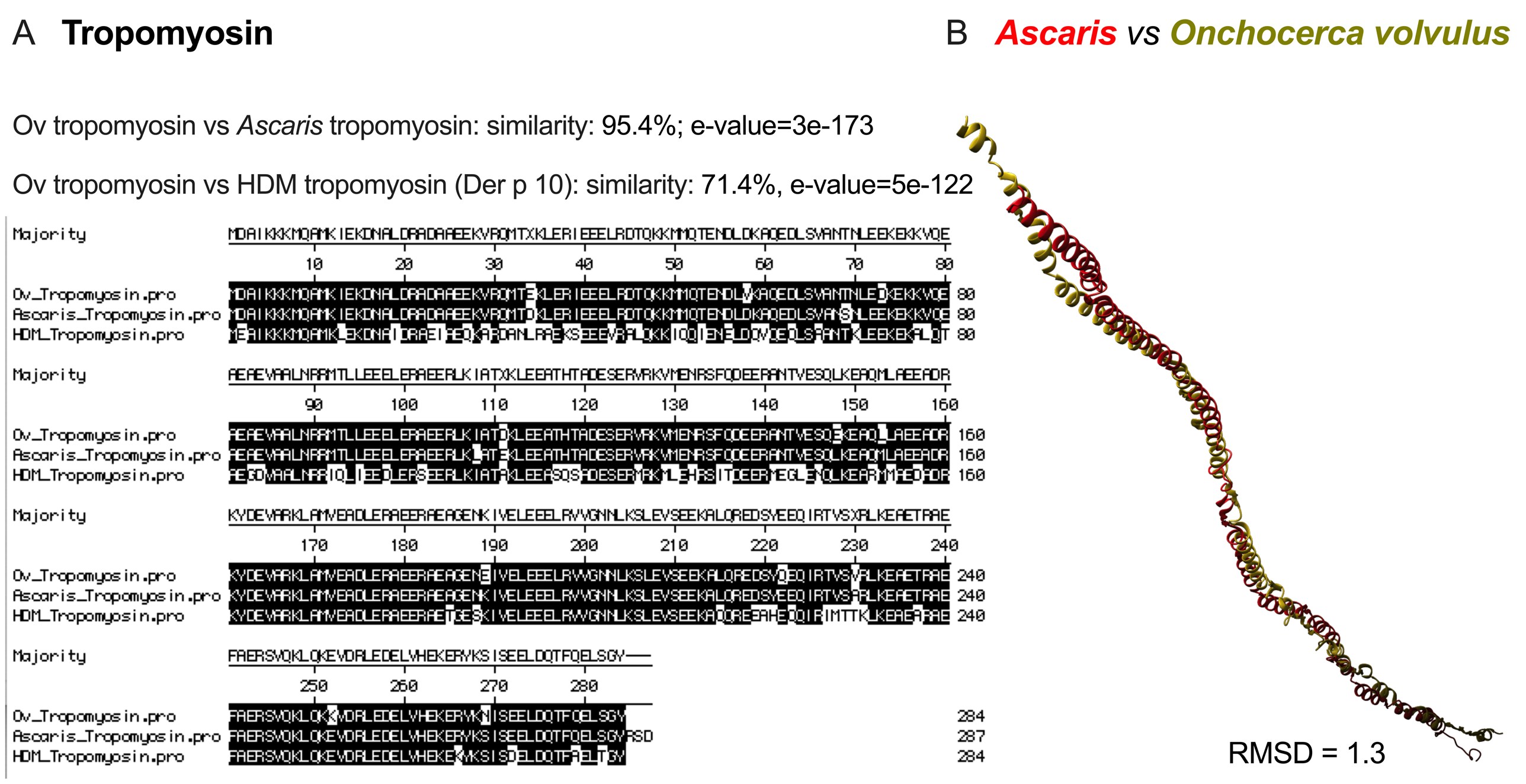

Supplement: S3 Fig — Alignment of Onchocerca volvulus, Ascaris and HDM tropomyosin (Der p 10) sequences showing identical (shaded in black) amino acids (A) and the predicted 2D structure of Onchocerca volvulus (beige) and Ascaris tropomyosin (red) for comparison (B). (TIFF) [file ppat.1009337.s003.tiff]

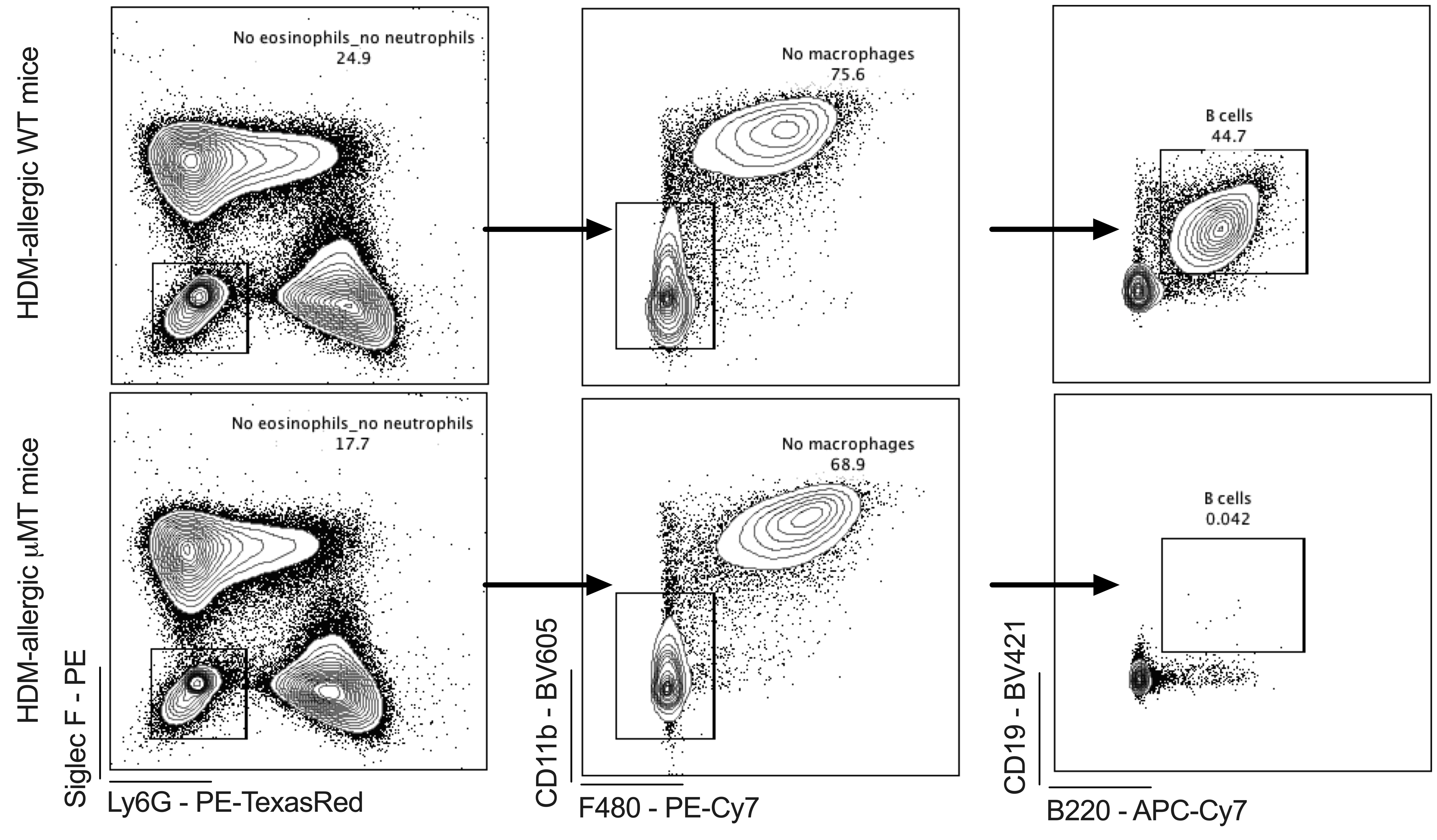

Supplement: S5 Fig — (TIFF) [file ppat.1009337.s005.tiff]
