# Supplementary material for: House dust mite sensitization drives cross-reactive immune responses to homologous helminth proteins
Source: PLoS Pathog. 2021 Mar 2;17(3):e1009337. doi: 10.1371/journal.ppat.1009337 (PMC7924806; doi:10.1371/journal.ppat.1009337)
Supplement: S1 Table — (DOCX) [file ppat.1009337.s006.docx]

**Supplemental Table 1: Flow cytometry antibodies**

| Antibodies | Source | Catalog # |
| --- | --- | --- |
| LIVE/DEAD Fixable Blue | Invitrogen | L23105 |
| PerCP-Cy5.5 anti-mouse CD4 (clone RM4-5) | BioLegend | 100540 |
| FITC anti-mouse CD19 (clone ID3) | BioLegend | 152404 |
| PE-Cy7 anti-mouse CD40L (clone MR1) | BioLegend | 106512 |
| BV785 anti-mouse CD44 (clone IM7) | BioLegend | 103059 |
| APC-Cy7 anti-mouse TCR-b (clone h57-597) | BioLegend | 109220 |
