# Supplementary material for: House dust mite sensitization drives cross-reactive immune responses to homologous helminth proteins
Source: PLoS Pathog. 2021 Mar 2;17(3):e1009337. doi: 10.1371/journal.ppat.1009337 (PMC7924806; doi:10.1371/journal.ppat.1009337)
Supplement: S2 Table — (DOCX) [file ppat.1009337.s007.docx]

**Supplemental Table 2: Flow cytometry antibodies**

| Antibodies | Source | Catalog # |
| --- | --- | --- |
| BV605 anti-mouse CD11b (clone M1/70) | BioLegend | 101257 |
| PE-Cy7 anti-mouse F4/80 (clone BM8) | BioLegend | 123114 |
| FITC anti-mouse IgE (clone RME-1) | BioLegend | 406906 |
| Alexa Fluor 700 anti-mouse CD63 (clone NVG-2) | BioLegend | 143924 |
| APC anti-mouse CD117 (c-Kit) (clone 2B8) | BioLegend | 105812 |
| PE anti-mouse FceR1a (clone MAR-1) | BioLegend | 134308 |
| PE-Tx red anti-mouse Ly6G (clone 1A8) | BD Horizon | 562700 |
| BV421 anti-mouse Siglec-F (e50-2440) | BD Horizon | 565934 |
| LIVE/DEAD Fixable Blue | Invitrogen | L23105 |
