# Supplementary material for: House dust mite sensitization drives cross-reactive immune responses to homologous helminth proteins
Source: PLoS Pathog. 2021 Mar 2;17(3):e1009337. doi: 10.1371/journal.ppat.1009337 (PMC7924806; doi:10.1371/journal.ppat.1009337)
Supplement: S3 Table — (DOCX) [file ppat.1009337.s008.docx]

**Supplemental Table 3: Flow cytometry antibodies**

| Antibodies | Source | Catalog # |
| --- | --- | --- |
| BV605 anti-mouse CD11b (clone M1/70) | BioLegend | 101257 |
| PE-Cy7 anti-mouse F4/80 (clone BM8) | BioLegend | 123114 |
| FITC anti-mouse CD11c (clone HL3) | BD Pharmigen | 553801 |
| V500 anti-mouse MHC-II (clone M5/114.15.2) | BD Horizon | 562366 |
| APC Cy-7 anti-mouse Ly6C (clone AL-21) | BD Pharmigen | 560596 |
| PE anti-mouse SiglecF (clone E50-2440) | BD Pharmigen | 552126 |
| PE-Tx red anti-mouse Ly6G (clone 1A8) | BD Horizon | 562700 |
| eFluor 450 anti-mouse CD19 (eBio 1D3) | Invitrogen | 48-0193-80 |
| LIVE/DEAD Fixable Blue | Invitrogen | L23105 |
